# Rare pediatric diseases and pathways to psychosocial care: a qualitative interview study with professional experts working with affected families in Germany

**DOI:** 10.1186/s13023-021-02127-2

**Published:** 2021-11-27

**Authors:** Stefanie Witt, Kaja Kristensen, Silke Wiegand-Grefe, Johannes Boettcher, Janika Bloemeke, Christina Wingartz, Monika Bullinger, Julia Quitmann, und die CARE-FAM-NET Studiengruppe

**Affiliations:** 1 Department of Medical Psychology, Center for Psychosocial Medicine, University Medical Center Hamburg-Eppendorf, Hamburg, Germany; 2 Department of Child and Adolescent Psychiatry, Psychosomatics and Psychotherapy, University Medical Center Hamburg-Eppendorf, Hamburg, Germany

**Keywords:** Pathway to care, Psychosocial care, Children with rare diseases, Qualitative study, Expert interviews

## Abstract

**Background:**

Rare diseases occur in early childhood and have a major impact on the quality of life of the affected children and their families. Their need for psychosocial support is considerable, but psychosocial care in Germany is still far from being part of routine care. We interviewed experts to explore how they describe the current pathways to psychosocial care, potential barriers and problems, and possibilities for improvements.

**Results:**

We conducted telephone interviews with 49 experts working in somatic medicine, psychosocial medicine, patient organizations, child and youth welfare, and the educational sector. Interviews were transcribed and analyzed using focused interview analysis. Results document ways of access and facilities used by families to receive psychosocial care. The barriers described by the experts can be summarized on three levels: the family-organizational level, the family-psycho-emotional level, and finally, the structural system level. Accordingly, suggestions for improvement were directed at these levels.

**Conclusion:**

Based on the experts' perspectives, there is ample room for improvement to facilitate the pathways to psychosocial care for children with rare diseases and their families. Unfortunately, there seems to be a long way to go before psychosocial care will be routinely provided. However, awareness of the issue among different professional groups is high, and numerous suggestions for improvement were made, including continuous expansion of services to all family members, strengthening of low-threshold services, simplifying application procedures, and more cooperation between different funding agencies as well as between different care providers.

## Background

Although rare diseases are rare in principle, their importance for public health is underlined by the high number of individual conditions whose combined population prevalence is estimated at 3.5–5.9% [1]. Hence, at any given time, an estimated four million people live with a rare disease in Germany [2], while 263–446 million people are affected worldwide [3]. About 70% of rare diseases have an exclusively pediatric-onset and are primarily of genetic origin [3]. Overall, they form a heterogeneous group of primarily complex clinical pictures, resulting in a reduced quality of life and/or life expectancy [4].

The diagnosis of a rare disease does not only affect the child but also parents and siblings. Therefore, rare diseases in children are often accompanied by a reduction in parental caregivers’ quality of life [5, 6]. After diagnosis, parents may experience this first period as a very stressful time and need to learn to cope with their child's rare disease [7]. In addition, many parents experience difficulties in receiving an initial diagnosis and finding an appropriate therapy resulting in enormous frustration [8]. These circumstances underline the need for psychosocial care for families of children living with a rare disease.

Studies show that the current support services do not always meet the families’ needs. For example, in a German survey assessing the psychosocial support needs of families with a chronically ill child, around one-third of parents named a specific need for psychotherapeutic intervention [9]. Furthermore, three-quarters of the parents mentioned needing support regarding legal and organizational matters [9]. Another German study focusing on families’ psychosocial situations with chronically ill children showed that one-third of the parents needed help for the whole family. However, only less than a third of them had previously been able to use family support services [10]. These results show that access to psychosocial care does not meet the specific needs of families with rare pediatric diseases and needs to be optimized accordingly. This insufficient access to psychosocial care for families of children with rare diseases is not only observed in Germany but worldwide [11–15].

Obtaining detailed information on pathways to care can help understand potential barriers and generate knowledge to adapt and improve psychosocial care delivery. A pathway to care is defined as a “complex intervention for shared decision-making and organization of care processes for a defined group of patients during a defined period of time” [16]. In research, experts have a unique position due to their detailed knowledge [17]. In this context, expert interviews can generate essential information about current problems, difficulties, and resources regarding psychosocial health care [18]. Therefore, this study aims to characterize the current pathways to psychosocial care for families of children with rare diseases from an expert perspective to identify components to improve access to psychosocial care.

## Methods

### Study design

The present study is part of the national multicenter project “Children Affected by Rare Disease and Their Families—Network (CARE-FAM-NET)" [19]. The project’s overall objective is to develop a cross-sectoral, sustainable psychosocial health care structure for children with rare diseases and their families. The aim is to learn more about the gap between medical and psychosocial health care for children with rare diseases and their families in Germany and to improve their mental health and quality of life in the long term. For this purpose, two new evidence-based psychosocial care interventions for children with rare diseases and their families are implemented and evaluated in a multicenter, randomized controlled trial. The project is conducted at 17 medical centers in 13 German federal states and will be transferred to routine care if the evaluation results confirm the participants’ benefits [20]. One secondary objective within this collaborative project focuses on examining the pathways to psychosocial care in more detail. Although rare diseases are very heterogeneous as a group, they have some commonalities, which is why it is worth considering rare diseases as a whole when thinking about pathways to psychosocial care. Living with a rare disease can be challenging for those affected in many ways. Despite differences in etiology and symptomatology, many rare diseases are chronic, associated with dysfunction in multiple systems, and require complex treatment that often has limited effectiveness [21]. In addition, many patients have difficulties accessing information, support, and care [22].

For this purpose, we conducted qualitative telephone interviews with experts, pediatric patients aged 8–21 years, and parents of pediatric patients aged 0–17 years to investigate current pathways to psychosocial care and barriers to develop an optimized pathway to care for families with children with rare diseases. The presented results are based on the interviews with the experts to describe in detail the perspectives and experiences of professionals. Nevertheless, these results should be considered preliminary as patients' and families' perspectives are yet not included.

This study presents the methods according to the Consolidated criteria for reporting qualitative research (COREQ) checklist.

### Participants and sampling

In this study, an expert was defined as a professional with extensive experience working with children with rare diseases and their families. Purposive sampling was used throughout the recruitment procedure to obtain a comprehensive and diverse sample [23]. The studied expert population covered a wide range of occupations for sufficient saturation. The planned sample size for the professions of psychosocial medicine, somatic medicine, patient organizations, child and youth welfare, and education was ten experts each, resulting in 50 experts. We recruited the experts via the authors’ networks and professional connections, internet search, and subsequent contact establishment. We obtained detailed information, informed consent, and institutional review board approval before the interviews were initiated.

### Data collection

We conducted semi-structured telephone interviews using an interview guideline to explore the following topics: (a) everyday life of children with rare diseases and their families, (b) known pathways to psychosocial care, (c) assessment of the adequacy of and access to psychosocial care and the referral practices of professionals, (d) psychosocial care recipients and situations in which psychosocial care is required and (e) barriers and suggestions for improvement of psychosocial care.

The first part of the interview guideline focused on socio-demographic and occupational data. The experts introduced themselves and described their daily work and previous experiences working with children with rare diseases and their families. The second part included questions on access to psychosocial care for children with rare diseases and their families. The third and final part included questions about specific barriers and obstacles to accessing care. During the interviews, the interviewer was able to modify the questions to make them more applicable to the specific interviewee, who was also allowed to discuss and explain any aspects that were not mentioned earlier in the interview.

We developed the interview guideline in an interdisciplinary team of experts in the field of rare diseases. The questions were selected and formulated according to international standards for qualitative research [24, 25]. Before starting the expert interviews, we also conducted several test runs using the think-aloud method to check the fit and comprehension of the questions [26].

Trained interviewers from the University Medical Center Hamburg-Eppendorf, Department of Medical Psychology, conducted the interviews via telephone using the interview guideline. In addition to the digital recordings, we transcribed and anonymized interviews. We used a standardized transcription method to ensure comparability between interviews.

### Data analysis

We analyzed the qualitative data using the focused interview analysis described by Kuckartz [27, 28]. This process included both inductive and deductive coding. First, we generated deductive categories based on the interview guideline. From that, we outlined a preliminary category system. Subsequently, we analyzed the transcripts by assigning text passages to the respective categories. We inductively created a new category or sub-category whenever a particular text appeared essential but could not be coded into an existing category. Finally, we re-analyzed 50% of all interviews and assigned all important passages to the previously created sub-categories to check and confirm the final code system. An independent second-rater analyzed a subset of randomly selected interviews, representing 20% of all interviews. We aimed to achieve an agreement between both raters of at least 70%.

## Results

### Sample description

Among the 111 experts contacted, 49 experts covering the five professional groups agreed to participate in the study (see Table 1). The participating experts were predominantly female (34 females vs. 15 males).

**Table 1:** Overview of interview partners and their field of work

Professional category	Number of professionals	Female	Male
Psychosocial medicine	10	6	4
Somatic medicine	10	6	4
Patient organizations	10	6	4
Child and youth welfare	9	8	1
Education	10	8	2
Total	49	34	15

The experts from the field of psychosocial medicine were currently working in (university) medical centers (n = 8) and outpatient counseling settings (n = 2). Their professions were psychologists (n = 4), physicians specializing in child and adolescent psychiatry (n = 3), pedagogues (n = 1), pediatricians (n = 1), and human geneticists (n = 1). Somatic experts were currently working in hospitals (n = 9) and a screening center (n = 1). These experts were neuropediatricians (n = 3), pediatricians (n = 2), pediatric surgeons (n = 2), pediatric endocrinologists (n = 1), and epidemiologists (n = 1). Experts from the category patient organizations worked in disease-specific patient organizations (n = 8), and organizations focused on patients with rare diseases in general (n = 2). Experts for the child and youth services category included respondents working in (outpatient) counseling (n = 3), (outpatient) care (n = 2), and kindergarten (n = 4). There were pedagogues and social workers (n = 4), kindergarten and preschool teachers (n = 4), and a nurse specialized in caring for pediatric patients (n = 1). Educational experts worked in specialized schools for children with special needs (n = 6) and schools caring for children with and without special needs (n = 4). These experts were teachers for children with special needs (n = 6), teachers (n = 3), and social workers (n = 1). All interviewed experts reported experiences working with children with rare health conditions. However, these experiences showed a huge variety regarding the duration of experiences [4 years to more than 25 years] and the type of diseases respectively diseases more broadly. Depending on the context, they were specialized in one group of diseases, offered support for children with chronic health conditions in general (Table 2).

**Table 2:** Overview of experts’ experiences in children with rare diseases

Area of experts’ experiences	n
Goldenhar syndrome	1
Morbus Fabry	1
Rett syndrome	1
Trisomy 21	2
Cognitive impairments	2
Congenital malformations	3
Endocrine disorders	3
Neurological disorders	2
Pediatric cancer	6
Short stature	1
Rare diseases	8
Chronic health conditions	10
Children with special needs	9

### Category system

The final code system includes seven main categories which are (1) pathways to psychosocial care; (2) psychosocial care facilities; (3) utilization of psychosocial care; (4) situations in which psychosocial care is required; (5) reasons for psychosocial care; (6) problems and barriers related to psychosocial care; and (7) suggestions for improvement related to psychosocial care, which comprises 48 sub-categories in total. The inter-rater agreement of the final code-system achieved an agreement of 74%, rating 20% (n = 10) of the interviews by an independent rater.

### Pathways to psychosocial care

The category “Pathways to psychosocial care” includes statements on the institutions and measures that initiate access to psychosocial care. Experts identify a wide range of entry points to psychosocial care concerning families' access to psychosocial care (Table 3).

**Table 3:** Pathways to psychosocial care

Sub-category	Description
Health care providers	General practitioners or hospitals can refer parents to suitable services
Self-research	Internet searches are an increasingly important pathway
Educational setting	Through regular contact with children and families, the need for psychosocial care often becomes apparent at an early stage
Social services	With rich networks, social services can be an important access point
Self-help groups/patient organizations	Exchange with other families helps to obtain an overview of available services

Experts describe parents’ self-research as one of the most important pathways to psychosocial care. In particular, parents would use information from the internet and social media. Especially experts from patient organizations mention that most families would find information about patient organizations via internet research.

Another path to psychosocial health care leads through patient organizations and self-help groups. The exchange with other families can help parents to find out which services are available and helpful. For example, a female expert from somatic medicine stated:

*“So the patients we look after, many of them have actually joined patient and self-help organizations and have found the groups through their own research, so to speak. And via the self-help groups a lot of information material is also passed on, ehm, including, of course, ‘where can I get ehm, help in the sense of psychosocial care?’”*

The experts report that families are also referred to psychosocial health care by their health care providers, such as general practitioners or physicians from clinics. These somatic experts are often the first persons talking to the parents, as they are already involved in communicating the diagnosis. According to somatic medicine experts, provision or referral to psychosocial care might depend on the diagnosis.

Educational experts describe that parents often turn to them when seeking psychosocial health care. As professionals from the educational sector often care for children over a long time, they can identify developmental delays or changes early. Another way for families to obtain information about psychosocial care providers is through social services.

### Psychosocial care facilities

The category “Psychosocial care facilities” describes different sectors where psychosocial care is offered and includes the different facilities where families receive psychosocial care. We distinguish between the medical and psychological sector and the social welfare and nursing sector (Table 4).

**Table 4:** Facilities providing psychosocial care

Sub-category	Description
Medical and psychological sector	Hospitals, social-pediatric centers, psychologists, or psychiatrists are facilities where families can receive psychosocial care
Social welfare and nursing sector	Social services, self-help groups, and patient organizations, church activities, rehabilitation centers, and respite care provide possibilities for families to receive psychosocial care

Children with rare diseases and their families tend to spend much time in hospitals. In some cases, hospitals are a focal point where families and children receive psychosocial support directly. Almost all experts mention so-called social-pediatric centers as essential facilities to receive psychosocial support. These centers are specialized, interdisciplinary outpatient facilities for children and adolescents and are distinguished by their multi-professional teams that provide both somatic and psychosocial care for the children. A female expert from the somatic field commented on the social-pediatric centers as follows:

*"Some of these [social-pediatric centers] have multidisciplinary teams, where psychologists are already involved. If a child is lucky enough to be treated in such a center, then the child is also well cared for psychosocially."*

However, families need a referral from registered specialists to receive treatment in these social-pediatric centers where only the affected child can usually receive psychosocial care.

Experts describe that families can also turn to psychiatrists or psychologists for psychosocial care. In addition, social services, support groups, patient organizations, church activities, rehabilitation centers, and respite care are described as other psychosocial-care facilities.

### Utilization of psychosocial care

The category “Utilization of psychosocial care” includes statements about which family members utilize psychosocial support and which characteristics are hypothesized to influence the utilization (Table 5).

**Table 5:** Utilization of psychosocial care

Sub-category	Description
Single parents	Single parents are often particularly burdened and in need of support
Child with rare disease	The need for psychosocial support often increases with the age of the child
Mothers	Within a stable relationship, mothers often make more frequent use of psychosocial support than fathers
Socioeconomic background	More personal and financial resources often facilitate access
Disease coping	Families who are coping well with the child's condition are perceived to be more willing to use support services

Experts report that single parents frequently use psychosocial care offers because they are often particularly burdened by high demands. However, couples also make use of these services. The experts observe that within a stable relationship, mothers use psychosocial support more often than fathers. According to the experts, the mother usually takes care of the affected child and its siblings. Many times, the mother manages everyday life and reduces working hours if needed. However, some experts perceive that fathers are increasingly involved in the care of their children and use psychosocial services more often than they did a few years ago. A male expert from the self-help sector made the following statement:

*"There is this long-standing idea that self-help is more of a women's issue, especially when it comes to rare diseases. So, in the sense that it is increasingly the mothers who become involved, who are more interested in rare diseases. That is still true in general. But I would say that fathers are also more involved, similar to the overall population of fathers who are becoming more involved in early educational issues."*

There is disagreement among experts as to whether psychosocial care depends on the parents' socioeconomic status. Experts who perceive an association between high socioeconomic status and utilization of psychosocial care use (n = 28) describe that parents with a higher educational background tend to search for information more independently and accept help more quickly in health deterioration situations because of more existing resources. These parents are more likely to know where to look for support and what to look for, are generally better off financially, and are thus able to travel greater distances to reach services. A female expert from self-help described her observation regarding the parents' occupation and the possibility to care for the affected child:

*It takes time to take care of everything. And those who have to work don't have that time. And often in families with a higher level of education and where the fathers have a better salary, the women, I have observed, can work part-time and take care of it. I also know someone who stopped working completely and then cared for the disabled child and the family. So that's the case with everyone where the man earns enough money. And those who really have to work or single parents, well, that's very difficult.*

Experts who do not perceive an association between service utilization and socioeconomic status (n = 12) observe that the existing strategies of the families to handle the child’s rare disease are more important than their socioeconomic background. Further, psychosocial care utilization also depends on the current family networks and coping strategies regarding worries and problems. Some experts assume that parents who cope well with their child's disease are also more willing to use support services than parents who find it challenging to accommodate the situation. For example, a male somatic medicine expert stated:

*So I wouldn't say that it [utilization of psychosocial care] depends on the social background, but more on whether the family has already dealt with it for a longer time and perhaps has become acquainted with similar cases.*

In addition, the experts report that the child concerned utilizes psychosocial support more often with increasing age. However, many children are unable to attend psychosocial care services due to cognitive and physical impairments.

### Disease-related situations in which psychosocial care is required

The category "Disease-related situations in wich psychosocial care is required" applies to statements from experts about the circumstances or situations related to the disease in which psychosocial support is most often sought. Although there is a consensus that professionals should inform families about psychosocial support services at every meeting, there are also specific situations in which families seek psychosocial support (Table 6).

**Table 6:** Disease-related situations in which psychosocial care is required

Sub-category	Description
Diagnosis	Parents often seek help once the diagnosis has been made
Disease coping	The phase where families have adjusted to the diagnosis and are ready to make use of support
Everyday life	When acute situations have been overcome, or significant everyday life changes are approaching
Progression and crises	Any type of progression or crisis, which might not even be related to the disease

A critical situation when families might need psychosocial support is at the time of the initial diagnosis. While some parents need time to collect their thoughts and understand what to expect before seeking help, other parents feel overwhelmed and need immediate help. According to experts, families also often seek psychosocial help during the process of coping. In this phase, families have already accepted their fate and are ready to receive help. Some families do not use psychosocial services until acute stress situations have subsided. These families use psychosocial health care to maintain or enhance family functioning and calm down. A female expert from psychosocial medicine assessed the situation as follows:

*My guess would be once the dramatic acute events are over. And so, the whole family tries to calm down a bit and catch its breath, so there is more space to focus on or look at ones’ mental health. Or maybe the family comes forward with more strength than in this situation where everything is on alert and in survival mode.*

Other situations in which families often use psychosocial support are everyday life changes, such as the entry to kindergarten or school or career orientation. At this time, parents have to clarify many organizational questions. Progressive courses of illness, extended hospital stays, or the child’s death are crucial situations in which parents often seek psychosocial support.

### Reasons for required support

The category "Reasons for required support" includes statements by the experts about reasons for seeking help. The statements are assigned to two categories: family matters, and information and organizational support (Table 7).

**Table 7:** Reasons why psychosocial care is needed

Sub-category	Description
Family matters	For all family members, situations requiring psychosocial support can arise for several reasons
Information and organizational support	Disease management, the organization of daily life, advice on legal and administrative issues, and exchange with other affected families are typical reasons for requesting psychosocial care

The experts describe that children with rare diseases often show behavioral changes. Parents are committed to taking all available measures so that their child can grow up as well as possible and learn to cope with his or her health condition. It is also essential for parents that other siblings do not suffer from their sibling’s health condition and receive sufficient attention and affection. According to the experts, a major concern for parents is to continue to provide adequate care for all children in times of deteriorating health. Experts perceive that parents often suffer significantly from the consequences of their child’s rare disease. Therefore, it is often helpful to talk about fears and worries with an external person and/or professional. A male expert from the field of somatic medicine described the following observations:

*"There is often the situation that they [the parents] say that they are already very burdened, that that it is a lot. They can't live their lives anymore. They have to give up their jobs.”*

Based on the interviews, experts identified four main reasons for organizational and informational support: assistance with disease management, daily life organization, advice on legal and administrative issues, and exchange with other affected families.

### Problems and barriers related to psychosocial care

According to the experts, numerous barriers prevent families from seeking and receiving psychosocial support. These barriers can be divided into three main categories: family circumstances, inadequate coping that prevents parents from using psychosocial help, and structural barriers (Table 8).

**Table 8:** Barriers in accessing psychosocial care

Sub-category	Description
Family circumstances	Parents may face a hectic everyday life and lack financial resources preventing them from accessing psychosocial care
Inadequate coping	Parents do not always realize that external help is needed. Prejudices can further intensify their reservations
Structural barriers	Low capacities as well as cultural and language barriers are frequent

According to experts, family circumstances with an often overloaded daily routine can be a barrier. Parents have to fit frequent physicians appointments, therapy sessions, and clinic visits into everyday life. Additional appointments for psychosocial care can be a challenge due to the lack of time. Another crucial barrier is a lack of financial resources. Only families with high socioeconomic status can afford services out-of-pocket that are not financed by health insurance. The statutory health insurance funds’ reimbursement of psychosocial care services is a frequently mentioned barrier in this context. While complex applications procedures may present a barrier to many parents, the health insurance does not always cover psychosocial care applied for. In addition, the number of approved therapy sessions may be limited.

The process of coping with the illness of a child is another barrier frequently mentioned by experts. Due to the often genetic origin of rare diseases, parents tend to blame themselves for their child’s disease. According to experts, the long process of accepting the child’s health condition is often accompanied by a lack of acceptance of specific therapies and psychosocial help. Families often do not recognize or acknowledge that they need support. Parents may have prejudices against psychosocial care and fear being stigmatized or perceived as weak because of their need for help.

The low capacity of psychosocial care provision is perceived as a major structural barrier to psychosocial care. According to experts, families usually receive adequate medical care, but it is often more coincidence whether they receive psychosocial care. On the one hand, there is a lack of psychosocial professionals with experiences in rare diseases. On the other hand, there is a lack of information among health care providers about whom families can turn to for psychosocial support. Besides, families often have to wait several months for appointments. One female somatic expert described the following barriers:

*Well, we often don't find a place, so we call different institutions, different practices, and we just don't get an offer. That's a common situation, or there are very long waiting times, or the insurance company says: "we don't cover it."*

Experts from all professional groups perceive language and cultural barriers of increasing relevance. Cultural and language barriers may limit communication between families and service providers. Additionally, families with a migration background often do not know the German health system in detail and which services are appropriate and included in the insurance. According to self-help experts, the cultural background can also influence how families deal with a rare disease and needs to be considered.

### Suggestions for improvement related to psychosocial care

Experts’ recommendations are divided into suggestions for improvement concerning the situation in the families, the organization of psychosocial care, and structural changes (Table 9).

**Table 9:** Recommendations to improve psychosocial care provision and access

Sub-category	Description
Families	Provision of information regardless of demand, extension to all family members, strengthening and expanding self-help and respite-term care
Organization	Simplification of the application process, more cooperation between different funding bodies, strengthening of low-threshold services especially in the outpatient sector
Structural changes	Strengthening the integration of psychosocial support in the health system and promoting more inter-professional cooperation and networking

The experts highlight the importance of constantly informing parents about potential support types that they are entitled to receive, and also advocate for expanding psychosocial care services for the entire family and not only for the affected child. They mention the benefits of strengthening and promoting self-help groups and patient organizations where parents’ can network and exchange information. Promoting respite care might be another approach to improve the situation for families. In many cases, respite care is not available or only for at least 24 h. However, as most parents experience a burden of care, they might benefit from a break and the chance to recover.

Regarding organizational improvements, experts recommend more simple application procedures. Many parents cannot submit the large number of application forms required to the responsible institutions in advance. Low-threshold procedures and more intensive cooperation between the funding agencies involved would be necessary to alleviate the application processes. Another recommendation is to expand psychosocial services in the outpatient sector and strengthen psychosocial care maintenance during the transition from inpatient to outpatient care and from childhood to adulthood.

Improving cooperation between the different health care providers and professions, strengthened by a single point of contact for psychosocial care, is one recommendation for structural changes made by the experts. Many experts prefer a dual structure of a team of somatic and psychosocial experts advising and accompanying families. A female expert from child and youth welfare explained the role of networking:

*Networking between the different partners, not only in the health system but across the sectors. So I think care, psychosocial care, the word psychosocial actually already implies it, is trans-sectional, which means that not only the medical sector with pediatrics and child and adolescent psychiatry and psychotherapy but also youth welfare and the various other systems involved. Yes, and this trans-sectional networking must somehow be better anchored in the system.*

Lastly, the interviewed experts emphasize a need for improvement in the provision of low-threshold services. Psychosocial health care has to be easily and quickly accessible. Information about services has to be formulated in simple language to be understandable for everyone. Both outpatient and inpatient settings should offer these low-threshold psychosocial health care services to all families with children with rare diseases.

## Discussion

The results of this study identified different pathways to care and facilities for psychosocial support, the characterization of family members and situations for which an increased utilization is assumed, and a description of barriers that corresponding measures proposed by the experts could address.

Our results revealed that provision or referral to psychosocial care is far from standard in treating rare pediatric diseases. Especially in somatic medicine, experts described that the pathway to psychosocial care often depends on the diagnosis and that the referral does not proceed equally well in all clinical areas. For example, psychosocial care in pediatric oncology is a standardized procedure [29], and for the experts, a successful example of the integration of psychosocial care into routine care. These descriptions show how important it is to strengthen psychosocial care so that it is not diagnosis-oriented but needs-oriented for the pediatric patients and their families.

Experts highlight the importance of self-initiated research as an essential access point to psychosocial care. Primarily via web searches, families can find information and access to psychosocial care. Patient organizations for specific rare diseases are a good starting point for initial research. If resources are available, patient organizations can also refer to psychosocial support services on their website. However, patient organizations do not exist for every rare disease. Thus, self-organized online communities, therefore, offer an alternative. Some experts even argued that information about the disease, which parents receive through online communities, may be more valuable than information they receive from physicians. Other studies have also shown that access via the internet has become increasingly important in recent years [30]. Through online communities, individuals experienced in dealing with their rare disease can eventually contribute to sharing information and even advise other families and health professionals [31]. Experts in this study emphasized that highly motivated families seek psychosocial support through this route, as it requires a high level of engagement.

Former studies already discussed that socially disadvantaged families often have more difficulties using psychosocial services due to an information deficit, financial hardship, health insurance, and reimbursement problems [12, 32–34]. These findings are supported by a number of experts in this study who argue that families with a higher educational level are more likely to seek appropriate support and make greater use of the services offered by self-help groups and patient organizations. The experts who consider families' socioeconomic background to be of secondary importance argue that it is much more important how well a family can cope with their child's health condition and how strong the family cohesion is. According to these experts, access to psychosocial support is often influenced by events that seem rather coincidental. This view is supported by a study that identified parental education level and family income to directly influence the help-seeking process. Instead, school personnel and the history of mental health service use play an important role in families' search for adequate psychosocial care [35].

The findings on situations of particular need for psychosocial care show that there are indeed specific situations such as the time of the initial diagnosis or resuming to everyday life, where the need and willingness to use psychosocial care offers may be heightened, which is also supported by other empirical findings [36–38]. However, the level of need and willingness to seek psychosocial care may vary from family to family, which makes the sustained provision of information and psychosocial care offers essential.

The barriers to accessing psychosocial care described by the experts related to the family situation (circumstances and coping) and how this can influence the utilization of services, and to factors at the level of the health care system that hinder the provision of psychosocial health care.

Studies that directly involve caregiving parents of chronically ill children illustrate the tremendous burden and distress they experience [9, 10, 39]. The experts in this study perceived that parents are often occupied with caregiving and administrative matters, so parents have no capacity for psychosocial care. In a survey on the psychosocial care of chronically ill children, in which 462 parents from Germany participated, the authors reported that almost 60% of parents feel overburdened with caring for the child and coping with their situation. Nearly 80% think that bureaucracy is a barrier to access psychosocial care [39]. The experts in this study emphasized that dealing with the disease is another major barrier for many families. Acceptance of the rare health condition is often accompanied by acceptance of the use of psychosocial services [36]. According to the experts, as long as parents do not accept their child's health condition, they often find it challenging to use further treatment. Experts saw possible solutions in the constant provision of information about psychosocial services, an expansion of services for all family members and not just the affected child, and the expansion of respite care. Furthermore, simplifying application processes and enhancing cooperation between funding agencies could help to overcome these barriers.

A barrier at the structural level that has increasingly challenged experts in recent years is the limited ability to accommodate families with a migration background, who have limited German language skills and less familiarity with the German health care system. This problem is not unique to rare diseases but is present throughout the health care system [40]. The need for low-threshold services and easily accessible and understandable information becomes particularly apparent here.

Another structural barrier described by the experts is a lack of capacities. Due to the lack of services, professionals often do not know where to refer families. This observation is reflected in the reports of parents caring for a chronically ill child, according to which 80% of the parents feel insufficiently informed about help for coping with their child’s health condition. The same survey shows that 65% of parents feel well informed about medical therapy options, but this is the case for only 25% of parents concerning measures to ease the burden on the family [39]. Results from the Rare Barometer Survey 2017 show similar results. Around 70% of respondents reported poor knowledge about their rights related to the consequences of the disease, financial support, and the relevant social services they are entitled to [41]. According to the experts, central contact points for psychosocial care and stronger multidisciplinary networking among professionals could be possible solutions.

It should be mentioned that much has also improved concerning multidisciplinary collaboration in recent years. In particular, establishing disease-specific and interdisciplinary centers for diagnosing and managing rare diseases has considerably enhanced the care situation [42]. Nevertheless, psychosocial care is not currently part of routine care in these centers. However, in the joint project CARE-FAM-NET, of which this study is a part, the aim is to implement cross-sectoral psychosocial care for children with rare diseases, their siblings, and parents and transfer it to routine care [19].

When processing and analyzing the expert recommendations, it was noticeable that they did not address some interesting aspects, such as their role as psychosocial care providers. For example, it is not clear from the interviews how one's behavior, e.g., asking about the family's well-being, can contribute to the provision of psychosocial support, or how best to handle a situation when the need for psychosocial support is identified but capacity to meet the demand is limited.

### Strengths and limitations

A major strength of this study is the large sample of experts with different professions so that a multi-perspective view was achieved. The use of expert interviews turned out to be an ideal approach to our research question as the subject area has not yet been sufficiently researched. Experts have profound knowledge that they can put it into the context of our research question [43].

A limitation of qualitative analysis is the reproducibility of the results [44]. How certain categories are structured and labeled can depend on the individual researcher and his/her position. Therefore, installing an inter-rater who works through a random interview selection using the category system is helpful. A reliability score can be regarded as a standard for the accuracy of the investigation, which represents a specific quality criterion [45]. The inter-rater agreement using the final code system achieved an agreement of 74%, which we consider sufficient.

Due to low response rates to interview requests, some cases made recourse to the authors' professional network. Some of the experts from the somatic and psychosocial fields were part of the CARE-FAM-NET project. This resulted in a pre-selection concerning the knowledge and basic attitude of the interviewed experts towards the research field and might have limited the diversity and breadth of the results. There was also a geographical clustering among the experts. The experts came mainly from the northern and western parts of Germany. It is therefore unclear whether the results are comparable across Germany.

Another limitation is that the presentation of results refers to the expert interviews only and therefore are preliminary. Only including all stakeholders, such as service providers and service users, can enable a balanced examination. A follow-up publication is planned when the data collection and analysis of the patient and parents interviews is completed to get a comprehensive view of current pathways and needs, allowing a broader perspective.

## Conclusions

Rare pediatric chronic health conditions pose an enormous challenge for all family members involved. The results of this qualitative study with experts of varying professions demonstrate that psychosocial care cannot yet be considered as part of routine care. The experts' statements clearly show that there is still ample room for improvement to facilitate access to psychosocial care. Suggestions for improvement by the experts to strengthen psychosocial care for families with children with rare diseases include, among others, continuous provision of information on psychosocial support, expansion of services to all family members, strengthening and expansion of patient-organizations, simplification of application procedures, and more cooperation between different funding agencies, strengthening of low-threshold services predominantly in the outpatient sector, strengthening the integration of psychosocial care into the health care system and promoting interdisciplinary collaboration and networking.

## Data Availability

The datasets generated and/or analyzed during the study are not available publicly. This was done to keep the interview transcripts confidential.
